# Dopaminergic and noradrenergic modulation of stress-induced alterations in brain activation associated with goal-directed behaviour

**DOI:** 10.1177/02698811211044679

**Published:** 2021-09-14

**Authors:** Peter van Ruitenbeek, Conny WEM Quaedflieg, Dennis Hernaus, Bart Hartogsveld, Tom Smeets

**Affiliations:** 1 Department of Neuropsychology and Psychopharmacology, Faculty of Psychology and Neuroscience, Maastricht University, Maastricht, The Netherlands; 2 Department of Psychiatry and Neuropsychology, Faculty of Health Medicine and Life Sciences, Maastricht University, Maastricht, Netherlands; 3 Department of Clinical Psychological Science, Faculty of Psychology and Neuroscience, Maastricht University, Maastricht, The Netherlands; 4 CoRPS – Center of Research on Psychological and Somatic Diseases, Department of Medical and Clinical Psychology, Tilburg School of Social and Behavioral Sciences, Tilburg University, Tilburg, Noord-Brabant, The Netherlands

**Keywords:** Dopamine, stress, goal-directed behaviour, neuroimaging

## Abstract

**Background:**

Acute stress is thought to reduce goal-directed behaviour, an effect purportedly associated with stress-induced release of catecholamines. In contrast, experimentally increased systemic catecholamine levels have been shown to increase goal-directed behaviour. Whether experimentally increased catecholamine function can modulate stress-induced reductions in goal-directed behaviour and its neural substrates, is currently unknown.

**Aim:**

To assess whether and how experimentally induced increases in dopamine and noradrenaline contribute to the acute stress effects on goal-directed behaviour and associated brain activation.

**Methods:**

One hundred participants underwent a stress induction protocol (Maastricht acute stress test; MAST) or a control procedure and received methylphenidate (MPH) (40 mg, oral) or placebo according to a 2 × 2 between-subjects design. In a well-established instrumental learning paradigm, participants learnt stimulus–response–outcome associations, after which rewards were selectively devalued. Participants’ brain activation and associated goal-directed behaviour were assessed in a magnetic resonance imaging scanner at peak cortisol/MPH concentrations.

**Results:**

The MAST and MPH increased physiological measures of stress (salivary cortisol and blood pressure), but only MAST increased subjective measures of stress. MPH modulated stress effects on activation of brain areas associated with goal-directed behaviour, including insula, putamen, amygdala, medial prefrontal cortex, frontal pole and orbitofrontal cortex. However, MPH did not modulate the tendency of stress to induce a reduction in goal-directed behaviour.

**Conclusion:**

Our neuroimaging data suggest that MPH-induced increases in dopamine and noradrenaline reverse stress-induced changes in key brain regions associated with goal-directed behaviour, while behavioural effects were absent. These effects may be relevant for preventing stress-induced maladaptive behaviour like in addiction or binge eating disorder.

## Introduction

Stress plays an important role in the development and maintenance of maladaptive behaviours like addiction (Brewer et al., 1998; Sinha, 2001; Sinha and Jastreboff, 2013). Particularly the hypothesis of stress-induced relapse has received substantial attention (Sinha, 2001; Sinha and Jastreboff, 2013), which has been linked to deficits in instrumental learning more generally (Schwabe et al., 2011), and reduced goal-directed behaviour, that is, increased *habitual* responding, specifically. Habitual behaviour has been described using a wide range of theoretical frameworks, including similarity based retrieval of episodic memory (Logan, 1988), contingency encoding models (Van Dessel et al., 2019) and dual-process models (Evans, 2008). A commonly shared characteristic of such frameworks is that habitual behaviour refers to actions/choices that are not driven by their current reward value, but rather by (rewarding) characteristics of the action/choice experienced in the past.

In individuals suffering from addiction, behaviour that was originally goal-directed has progressively become more habitual and may dominate the behavioural repertoire; that is, behaviour is no longer sensitive to outcome value and is assumed to be no longer under cognitive control (Everitt and Robbins, 2005, 2016). In the context of stress, a similar shift in instrumental learning can be observed in the form of rigid and computationally frugal habits in lieu of flexible yet cognitively demanding goal-directed behaviour (Goldfarb et al., 2017; Quaedflieg et al., 2019; Schwabe and Wolf, 2009, 2010; Smeets et al., 2019; for reviews see Schwabe and Wolf, 2013; Schwabe et al., 2010b; Wirz et al., 2018).

The neural correlates of goal-directed behaviour involve a wide range of regions associated with reward value (e.g. orbitofrontal cortex; Graybiel and Grafton, 2015; Quaedflieg et al., 2019; Valentin et al., 2007), instrumental behaviour (e.g. caudate and putamen; de Wit et al., 2012; Tricomi et al., 2009; Watson et al., 2018) and cognitive control (e.g. anterior cingulate; Holroyd and Yeung, 2012). In individuals suffering from addiction, a lack of goal-directed behaviour is associated with *increased* activation of brain regions underlying habitual behaviour (e.g. dorsal striatum) and *decreased* activation of brain regions underlying goal-directed behaviour (e.g. ventral striatum and frontal cortex), compared with healthy controls (Belin et al., 2013; Vollstädt-Klein et al., 2010). This ventral-to-dorsal activation shift might reflect a neurobiological mechanism associated with stress-induced relapse. Indeed, acute stress is associated with a shift in brain activation from ventral-to-dorsal striatum, and an impaired outcome processing in prefrontal cortex can be observed too (e.g. Arnsten, 2009a; Dedovic et al., 2009; Schwabe, 2017; Schwabe et al., 2012).

It is thought that catecholamines such as dopamine and noradrenaline are crucially involved in goal-directed behaviour and habit formation (Gasbarri et al., 2014; Nelson and Killcross, 2013; Schwabe et al., 2010a). First, dopamine is known to signal positive reward prediction errors (Maes et al., 2020; Sharpe et al., 2020) and catecholamines more generally underlie motivational processes associated with approach behaviour (Berridge and Robinson, 2016; Bouret et al., 2012). Second, reductions in brain-wide catecholamine availability through acute phenylalanine/tyrosine depletion increased the habitual behaviour in healthy female participants (de Wit et al., 2011b). Similarly, increased catecholamine availability by administering L-DOPA enhanced model-based control (akin to goal-directed behaviour) (Wunderlich et al., 2012), particularly in individuals with high working memory capacity (Kroemer et al., 2019), suggesting that catecholaminergic involvement in goal-directed behaviour might involve modulation of frontal cortex functional capacity. In contrast, de Wit et al. (2011a) did not observe differences in goal-directed behaviour between Parkinson’s disease patients on- and off-dopamine agonist medication (L-DOPA). However, the patients on medication also showed higher disease severity, which was associated with worse performance. Third, such modulation of cognitive control and frontal cortex functional capacity has also been reported for catecholamine enhancement using methylphenidate (MPH) both in healthy volunteers (Pauls et al., 2012) as well as in individuals with attention deficit hyperactivity disorder (Rubia et al., 2014).

Catecholaminergic involvement in goal-directed behaviour is also relevant in the context of stress, relapse and addiction. Acute stress exposure is associated with increased cortical and striatal dopamine release, both in humans (ventromedial prefrontal cortex (vmPFC): Lataster et al., 2011; Pruessner et al., 2004, for review see: Vaessen et al., 2015) and rodents (e.g. Jackson and Moghaddam, 2004; King et al., 1997; Tidey and Miczek, 1996). Stress induced dopamine release is likely the end-result of increased phasic firing of ventral tegmental area (e.g. Holly and Miczek, 2016; Ungless et al., 2010) and locus coeruleus (Kawahara et al., 2000; Valentino and Van Bockstaele, 2008) neurones, two regions with the highest density of dopamine and noradrenaline neurones, respectively. Moreover, stress and rewards engage the same populations of ventral tegmental area dopamine neurones (Del Arco et al., 2020), and stress-induced dopamine release may trigger relapse-like behaviour in animal models of addiction (Wang et al., 2005, 2007). Thus, importantly, these studies indicate that stress-induced shifts in goal-directed behaviour may involve catecholaminergic mechanism that are typically activated by acute stress and that may contribute to stress-induced relapse. However, a definitive demonstration of the role of catecholamines (specifically, dopamine and noradrenaline) in orchestrating a shift in goal-directed behaviour under stress is currently lacking.

Here, we aimed to unravel the functional role of catecholamines in the effect of acute stress on goal-directed behaviour and associated brain activation. One hundred healthy participants underwent a stress-induction protocol, or a control procedure (Maastricht acute stress test (MAST); Smeets et al., 2012), and received oral methylphenidate (MPH) to increase synaptic catecholamine levels, or placebo (PLC), after which we assessed goal-directed behaviour and associated brain activation using a previously validated instrumental learning paradigm in combination with outcome devaluation (Hartogsveld et al., 2020). Based on previous work (Fournier et al., 2017; Hartogsveld et al., 2020; Schwabe and Wolf, 2010; Smeets et al., 2019), we expected that acute stress would reduce goal-directed behaviour, and that this would be reflected in the corresponding neural activation (e.g. increased activation in the dorsal striatum and putamen, decreased activation in the orbitofrontal cortex and anterior cingulate cortex). In light of the well-known enhancing effects of MPH on dopamine and noradrenaline release (Volkow et al., 2002), we also expected MPH to increase habitual behaviour at the expense of goal-directed behaviour. Most importantly, however, we expected that administration of MPH would modulate the effect of acute stress on goal-directed behaviour and associated neural activity (i.e. a stress-by-MPH interaction), thereby providing evidence for catecholaminergic regulation of goal-directed behaviour under stress.

## Materials and methods

### Participants

All participants were recruited and tested between January 2017 and May 2019. Healthy male and female young adults were recruited from the general population. Conservative power analyses (GPower; Faul et al., 2007: α = 0.05 two-tailed; 1 − β = 0.80) based on previous effects size of MAST observed in our previous studies in a 2 (MAST: stress and control) × 2 (Value: valuable, devalued) repeated measure design (η^2^_p_ = 0.10; Smeets et al., 2019), indicate that the required sample size is 80 participants. One hundred participants entered the study (56 female, *range* 18–35, $\bar{x}$ = 22.47 years, SE = 0.34) after having provided written informed consent and following a medical examination. Potential participants received a full physical examination to determine their suitability. First, exclusion criteria were assessed by means of a questionnaire. Participants were excluded in case of regular intoxications (i.e. substance/drug use in the 3 weeks prior, >24 alcoholic units per week), Body mass index outside the range of 18–28 m^2^/kg, pregnancy, the presence of non-removable metal objects in or on the body, and current or past medical condition. Subsequently, a standard physical medical examination, including a reassessment of the medical questionnaire, assessment of vital signs, electrocardiogram, drug screen, blood biochemistry, haematology, serology and urine-analysis, was performed by a licenced, independent physician. Any abnormalities in the results of the medical examination were evaluated at the discretion of the physician and if indicative of a medical condition (e.g. blood pressure diastolic >90 mmHg, systolic >140 mmHg, renal, pulmonary, gastrointestinal, cardiovascular, hepatic, psychiatric or neurological disease/disorder), participants were excluded from further participation. Upon full participation, participants received financial compensation for their time investment. Of the 100 participants that entered the study, data from two participants were removed due to high baseline cortisol levels (i.e. >3 SD), three participants discontinued due to personal reasons, one participant exhibited chance-level performance on the stimulus–response–outcome (S–R–O) associations (i.e. <50% correct on the final assessment during training and reminder phase), and one due to technical reasons. The study was approved by the local Medical Ethics Committee Academic Hospital/Maastricht University (nr.METC163021) and conducted in accordance with the Declaration of Helsinki and its amendments (World-Medical-Association, 1964, 1996, 2008, 2013).

### Experimental design and manipulations

The study was conducted according to a 2(drug: MPH vs. PLC) × 2(MAST: stress vs. control) between-subjects partially blind design. Both participant and experimenter were blind with respect to drug administration. For practical reasons, the experimenter guided the whole test day and administered the MAST, thus the experimenter was not blind to the MAST condition. Participants were randomly allocated to one of the conditions using a computerised block-randomisation procedure taking sex and age into account for equal distribution across conditions, which resulted in the following groups: stress/MPH (*n* = 24, 14 female, $\bar{x}$ = 22.25 years, SE = 0.63), stress/PLC (*n* = 21, 15 female, $\bar{x}$ = 22.81 years, SE = 0.65), control/MPH (*n* = 24, 14 female, $\bar{x}$ = 21.37 years, SE = 0.52), control/PLC (*n* = 24, 14 female, $\bar{x}$ = 23.83 years, SE = 0.88).

#### Stress

**Figure 1.**
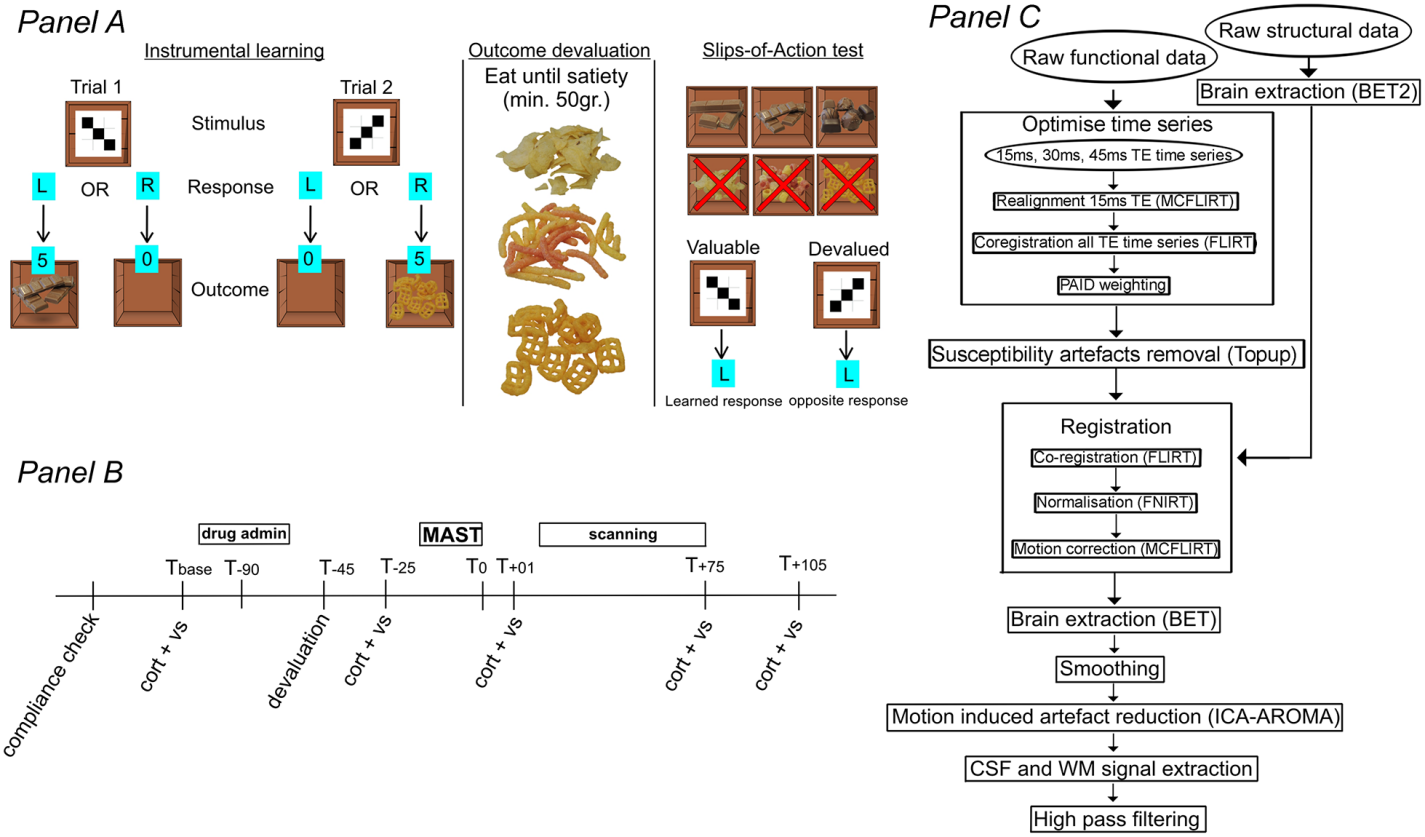
Overview of the instrumental learning task, procedure and imaging processing pipeline. Panel (a) Overview of the instrumental learning task. Upon presentation of a stimulus, participants responded with a left hand or right hand button as fast as possible. If a ‘correct’ response was provided, a box opened and a virtual reward was inside (chocolate or crisp; 75% contingency) and points were collected. All ‘Incorrect responses’ resulted in an empty box and no points. Explicit S–R–O association knowledge was assessed after every two blocks. After each block, participants received a small snack (chocolate and crisp) to incentivize learning. For the slips-of-action phase, devalued rewards with a red cross superimposed on an image of potential outcomes were shown before every block. Panel (b) Day 2 overview. Vital signs and saliva samples were collected at baseline (*T*_base_), post-devaluation (*T*_−25_), post-MAST (*T*_+01_), post-scanning (*T*_+75_), and at the end of the test day (*T*_+105_). *T*_x_: time in minutes relative to the end of the MAST; cort + vs: salivary cortisol sample and vital signs collection; drug admin.: administration of MPH 40 mg orally or placebo. Panel (c) fMRI processing pipeline. BET: brain extraction tool; CSF: cerebrospinal fluid; FLIRT: FMRIB linear registration tool; FNIRT: FMRIB nonlinear registration tool; ICA-AROMA: independent component analysis-based automatic removal of motion artefacts; MCFLIRT: motion correction; PAID: parallel acquired inhomogeneity desensitisation; TE: time of echo; WM: white matter.

The MAST (Smeets et al., 2012) was used to induce acute stress and is a reliable method to induce strong autonomic, glucocorticoid and subjective stress responses (Quaedflieg et al., 2017). The MAST combines physical stress induction, unpredictability, uncontrollability and social-evaluative nature of other stress induction protocols. In short, participants alternated between putting their hand in 2°C water for a period between 45 and 90 s and doing mental arithmetic (counting back from 2043 in steps of 17) while their faces were recorded and social-evaluative pressure (i.e. negative feedback) was provided by an experimenter unfamiliar to the participant. The control procedure was similar to the experimental procedure with the difference that water was lukewarm (36℃) and participants had to count from 1 to 25 at their own pace while no social pressure was applied. To determine individuals’ responses to the stressor, salivary cortisol samples and vital signs (heart rate (HR), systolic (SBP) and diastolic (DBP) blood pressure) were obtained prior to and following the MAST (see Figure 1 panel (b)). Subjective stress was assessed after performance of the MAST using visual analogue scales (VAS). Participants placed a vertical mark on three 10 cm horizontal lines indicating how they felt at that moment. Anchors were ‘not at all stressful’, ‘extremely stressful’; ‘not at all painful’, ‘worst pain imaginable’; ‘extremely pleasant’, ‘extremely unpleasant’.

#### Neuroendocrine stress response

Salivary cortisol was used to assess the neuroendocrine stress response and was collected through synthetic Salivette (Sarstedt, Etten-Leur, The Netherlands) devices. Saliva samples were stored at −20°C immediately after collection and kept until data collection was completed. Salivary cortisol levels were determined in singlet using a chemiluminescence immunoassay with high sensitivity (IBL International, Hamburg, Germany). This method produces intra- and inter-assay coefficients for cortisol below 9%. Concentration values at indicated time points are in reference to the end of MAST (see Figure 1 panel (b)). Next to these concentration values, areas under the curve were calculated by AUCg = (((*T*_base_ + *T*_−25_)/2) × 90) + (((*T*_−25_ + *T*_+01_)/2) × 26) + (((*T*_+01_ + *T*_+75_)/2) × 76) + (((*T*_+75_ + *T*_+105_)/2) × 30) to obtain a measure of total cortisol and AUCi = (AUCg) − (*T*_base_ × (90 + 26 + 75 + 105)) to obtain a measure for cortisol increase (Pruessner et al., 2003).

#### Methylphenidate

MPH (40 mg, oral) was administered to increase synaptic dopamine and noradrenaline levels in the brain during task performance in the MR scanner (i.e. 105 min after administration). Orally administered MPH immediate release formulation reaches *C*_max_ between 60 and 120 min after administration, occupies approximately 70% of striatal dopamine transporters and plasma levels decrease to 50% after approximately 6 h (Volkow et al., 2002). Existing data suggest a wide margin of a safe dose–response range 10–90 mg (Mehta et al., 2000; Volkow et al., 2002). MPH’s actions on extracellular dopamine and noradrenaline levels among others involve blockade of dopamine and noradrenaline transporters in the striatum and frontal cortex (Montgomery et al., 2007). Furthermore, MPH has been shown to increase cognitive control (Pauls et al., 2012; Rubia et al., 2014), which is crucial for successful goal-directed behaviour. Both MPH and acute stress are associated with changes in ventral tegmental area and locus coeruleus cell firing (e.g. Holly and Miczek, 2016; Karim et al., 2017), suggesting a degree of overlap in neurochemical mechanisms associated with modulation of goal-directed behaviour under stress and in response to stimulants.

#### Instrumental learning task

A four-stage instrumental learning task administered across 2 days was used to assess goal-directed and habitual behaviour (Smeets et al., 2019, see Figure 1 panel (a)). Participants learned six S–R–O associations by trial-and-error on day one (stage 1). Visual stimuli consisted of abstract black and white block figures in a 3 × 3 grid on the outside of a box to which participants pressed a left- or right-hand button as fast as possible. From a collection of eight chocolate and eight crisps type rewards presented as images on a single A4-sized paper, participants selected six preferred food type outcomes beforehand (three chocolate and three crisp) that served as rewards/outcomes. Following a ‘correct’ response, the box opened, and both a virtual reward (chocolate or crisp type reward) and points (ranging from 5 to 1, depending on reaction time) were presented. ‘Incorrect’ responses lead to an empty box and no points. Left/right button presses were associated with the optimal outcome for 3/6 stimuli. A contingency rate of 75% was implemented; that is, in 25% of all correct button presses no reward was presented and no points were collected. In the learning stage, participants completed eight blocks of 24 trials, totalling 192 trials. After each block of trials, participants consumed a small snack (chocolate or crisp) to make the receipt of rewards ‘feel’ more realistic, and to incentivize learning of the S–R–O associations, explicit knowledge of which was assessed after every second block of trials. Participants were instructed to collect as many rewards and points as possible.

On day 2, participants performed a reminder task with the same S–R–O associations (stage 2, two blocks of 24 trials), after which explicit knowledge of the S–R–O associations was assessed. Following the reminder task, one reward type (crisp or chocolate) was selectively devalued (stage 3). Fifty grams of chocolate or crisps were initially presented, which participants were required to eat. A subsequent 200 g were presented, and participants were urged to eat as much as possible until satiety was reached. The number of grams eaten by the participant was recorded. Following this devaluation approach, two 100 mm VASs measured the extent to which participants felt hungry (anchors: not at all hungry–very hungry) and felt like eating something tasty (anchors: not at all–very much so). Distance from the start of the line to the markings were measured and converted to a percentage of the total line.

Finally, the slips-of-action (SOA) task (six blocks of 24 trials, totalling 144 trials) was performed in the MR scanner (stage 4; day 2). For non-devalued outcomes, participants were instructed to press buttons associated with presentations of a food reward; for stimuli that were associated with food rewards that participants sampled until they were satiated in stage 3 (i.e. devalued outcomes), participants were instructed to press the *opposite* button (compared with previously learnt actions). Within the context of our paradigm, opposite button presses for these particular stimuli are considered goal-directed responses since they are indicative of an action associated with avoidance of the devalued reward and is in accordance with the new instructions that contrasts the presumed dominant learned response. In addition to appetitive devaluation of the rewards during stage 3, we aimed to cognitively devalue the outcomes during stage 4 by showing images of all outcomes at the start of every block of trials, and selectively superimposing a red cross on the devalued outcomes. Learned responses served as the primary outcome measure, which were defined as the response to a stimulus that leads to a valuable or devalued outcome (i.e. a food reward). Feedback was not provided during stage 4 to prevent relearning of associations. Inter-trial-intervals were jittered by applying a variable interval between 6 and 10 s following stimulus offset.

### Procedures and testing

Participants were instructed on each day of the 2 day procedure to visit our facilities well rested, not having performed any strenuous exercise within the previous 24 h, not having used any over-the-counter drugs in the past 2 days or prescribed drugs in the past 3 weeks, not having consumed alcohol since 19.00 h. the day before or caffeine-containing products or food the last 3 h, or smoked during the last 2 h. No participant reported any violations of these requirements upon enquiry at the start of the test day. On day 1, participants learned the S–R–O associations. On day 2 (i.e. the following day) participants received either oral capsules of PLC or MPH (*T*_−90_ relative to end of MAST) that had to be swallowed whole assisted by plain water. Selective devaluation (*T*_−45_) was achieved by having participants eat until satiety. At *T*_−15_ the MAST was performed (between 13.30 or 14.30 h to avoid high cortisol levels observed during morning hours) where after subjective levels of stress, pain and unpleasantness were assessed. MR scanning (*T*_+15_) was performed lasting 1 h. Participants performed the SOA during the peak cortisol and MPH levels. Structural, resting state brain activation and arterial spin labelling images were also recorded (all to be reported elsewhere). Finally, three cognitive tasks were performed typically sensitive to frontal lobe dysfunctions (i.e. N-back task, stop signal task, and Iowa gambling task; all to be reported elsewhere).

#### Imaging data acquisition and processing

Functional imaging was performed using a Siemens 3-Tesla Prisma MRI scanner. Each volume consisted of 42 slices, consisting of 3 mm isotropic voxels in a 224 mm field of view. Slice thickness was 3 mm with no gap between the slices. TR = 1000 ms and a multi-echo sequence was used to optimise the signal for each voxel offline (TE 1 = 5 ms; TE 2 = 29.93 ms; TE 3 = 44.86 ms). Flip angle was 60° and a multi-band acceleration factor of three was used. For co-registration, high-resolution T1-weighted structural images were obtained using an MPRAGE sequence resulting in 256 slices and 0.7 mm isotropic voxels in a 224 mm field of view. TR = 2400 ms, TE = 2.34 ms and flip angle = 8°.

Analyses were performed using custom and FMRIB’s Software Library (FSL; Jenkinson et al., 2012) scripts (see Figure 1 panel (c) for an overview). First, images from the three echo data were combined to construct a single optimised 4D image in which the TE with the best signal-to-noise ratio was determined and used per voxel. Therefore, realignment was performed using motion correction FMRIB linear registration tool (MCFLIRT) (Jenkinson et al., 2002) for the first echo data and applied to other two echo data by registering them using FLIRT (Jenkinson and Smith, 2001; Jenkinson et al., 2002). Next, parallel-acquired inhomogeneity desensitisation (PAID; Poser et al., 2006) weighting was performed by splitting the first 30 volumes from the time series and minimally smoothing them using a 2 mm FWHM Gaussian kernel to assist in PAID weight estimation. Of the 30 volumes a mean image and standard deviation were calculated, and the mean was multiplied by the echo time and divided by the standard deviation. Values were then adjusted such that the value per voxel is 1. Then, individual images are divided by the sum of the images and the weights are applied to the individual echoes. Finally, the weighted echoes are summed to generate a weighted time series. The same processing was applied to reverse phase encoded acquired images with distortions in opposite directions and which were used to determine susceptibility-induced off-resonance field to correct for the distortions using FSL’s top up (Andersson et al., 2003; Smith et al., 2004). These PAID weighted images were then subjected to a pre-processing pipeline using FSL6.0. The pipeline included brain extraction of the anatomical images using brain extraction tool (BET2; Smith, 2002) assisted by using coordinates of the massa intermedia for accurate extraction and manual parameter setting for each brain for high quality results.

Functional data were further pre-processed using FSL FMRI expert analysis tool (FEAT) and consisted of the following steps: removal of the first three volumes of the functional data, a four pass rigid-body motion correction (MCFLIRT; Jenkinson et al., 2002), co-registration of the functional data with the anatomical data using FLIRT and normalisation to MNI space using FNIRT (Andersson et al., 2010). The pre-processed functional data were inspected manually for exceedance of motion limits (framewise displacement >1 mm), which none of the participants exceeded. Subsequently the data were smoothed with a 5 mm FWHM Gaussian kernel, and independent component analysis-based automatic removal of motion artefacts (ICA-AROMA) (Pruim et al., 2015) was applied to remove head motion related noise. ICA-AROMA is an automatic procedure that uses independent component analysis to identify components representing head motion generated noise. Subsequently it removes the components from the data using least squares regression. Next, data were high pass filtered (>0.008 Hz). Finally, to account for signal from white matter and cerebrospinal fluid (CSF), white matter and CSF masks were created by segmenting the anatomical data. The masks were co-registered with the functional data using the inverse of the previously created transformation matrix and used to extract the signal from the time series.

### Neuroimaging data analysis

For one participant data were lost due to a technical error. Therefore, imaging data were analysed for 92 participants. For every participant a statistical model of the BOLD response was constructed that consisted of regressors for baseline epochs, Valuable_correct_ (action associated with a non-devalued outcome), Valuable_incorrect_ (action associated with avoiding a non-devalued outcome), Devalued_correct_ (action associated with avoiding a devalued outcome), Devalued_learned-response_ (action associated with a devalued outcome), white matter and CSF signal averaged over voxels, and first derivatives. Subsequently, the BOLD signal associated with two trial types (Valuable_correct_ and Devalued_learned-response_) was contrasted with the BOLD signal associated with baseline epochs. Baseline epochs for every trial were defined as the final time period of the presentation of the inter-trial fixation cross following the presentation of the stimulus. The duration of the baseline epoch for every individual trial was the same duration as the reaction time (RT) on the previous trial. Therefore, every trial epoch is compared with a baseline epoch of identical duration, but never overlapped. To determine significant trial-baseline activation differences, FEAT (Woolrich et al., 2001, 2004) was used with a *p* < 0.05 family wise error (FWE) corrected as threshold and threshold free cluster enhancement (TFCE) applied. The resulting contrast parameters of estimate (COPEs) were normalised to MNI space (FNIRT) and merged into a 4D image. A second level analysis was performed in which the interaction between MAST and drug was determined on activation representing goal-directed behaviour (Valuable_correct_ − Devalued_learned-response_), i.e. trials in which the participant erroneously responded with a learned response to a devalued outcome (Devalued_learned-response_) contrasted with trials in which participants correctly responded with a learned response to a non-devalued outcome (Valuable_correct_). For this contrast, positive values suggest greater activity for valuable outcome trials, while negative values indicate greater activity for Devalued_learned-response_ trials.

A drug-by-MAST interaction was assessed within a mask of whole brain grey matter with permutation testing using FSL’s randomise with 10,000 permutations and *p* < 0.01 FWE-corrected as threshold and TFCE applied. Significant clusters were determined by thresholding the resulting statistical map using a minimum cluster size of 25 voxels. To further determine how MPH modulates MAST-induced brain activation associated with (reduced) goal-directed behaviour, follow-up analyses of the interaction were performed to assess the effect of MAST separately in PLC and in MPH groups using the same permutation testing procedure (*p* < 0.05, FWE corrected, TFCE applied). The simple main effect of MAST in PLC was specified by stress < control and stress > control *t*-contrasts indicating less and more activation in the stress condition, respectively. Finally, to further characterise the interaction and detect potential modulation of the effect of stress by MPH, conjunction statistical maps were formed between the *p*-values statistical maps associated with *t*-contrasts for stress > control and for the bidirectional F-test of MAST in the MPH-groups, and between the *p*-values statistical maps associated with stress < control *t*-contrast and bidirectional F-test of MAST in the MPH-groups (cluster threshold 25 voxels). Beta values for Valuable_correct_ _−_ Devalued_learned-response_ in statistically significant clusters were extracted and displayed.

In the main text, we confine our results to regions previously associated with instrumental behaviour (de Wit et al., 2012; Tricomi et al., 2009; Valentin et al., 2007; Watson et al., 2018), that is, orbitofrontal cortex (OFC), vmPFC, anterior cingulate (ACC), paracingulate, premotor cortex (PMC), middle temporal gyrus, putamen, caudate, operculum, amygdala and insula, as determined using Harvard-Oxford cortical structural, Harvard-Oxford subcortical structural and Jeulich Histological atlases as implemented in FSLeyes, FSL6.0.3. A complete overview of the drug-by-MAST interaction on brain activation is reported in the Supplemental Material (Table S2).

Finally, for significant clusters average COPE values for Valuable_correct_ − Devalued_learned-response_ were correlated with a devaluation sensitivity index (DSI: e.g. Watson et al., 2018) defined as the difference between percentage learned response on valuable and devalued trials for every participant-group separately. Positive correlations between positive difference in activation (Valuable_correct_ − Devalued_learned-response_) and DSI is interpreted as that brain area being associated with the tendency to respond more for valuable outcomes compared with devalued outcomes. Only participants with an SOA score of at least 1 were considered for this analysis (*n* = 82). Given the skewed distribution of the Valuable_correct_ − Devalued_learned-response_ Spearman’s correlation was used and Bonferroni’s multiple comparisons correction was applied per group (*p* = 0.05/11, α = 0.005).

### Data and statistical analysis

Behavioural data were checked for outliers (±3SD) and non-normality using the Shapiro-Wilk tests and transformed by taking the natural log of the values whenever needed. Presence of outliers in the number of learned responses in the instrumental learning task were determined per condition (stress, drug and value), per block (1–6). No outliers in the number of learned responses were detected. As there were no significant differences between blocks and the blocks did not interact with any other factor, all blocks were concatenated by averaging the scores over blocks. *α* < 0.05 was regarded as statistically significant. In case of violations of the sphericity assumptions as shown by significant Mauchly’s test, Greenhouse–Geisser corrected values are reported. For all significant analyses of variance (ANOVAs) partial eta squared (η^2^_p_) are reported as a measure of effect size (Fritz et al., 2012).

MAST (stress and control), drug (MPH and PLC) and time (*T*_base_, *T*_−25_, *T*_+01_, *T*_+75_ and *T*_+105_) effects on cortisol levels and physiological stress measures (HR, SBP and DBP) and the interaction between these factors were assessed using a repeated measures ANOVA with time as repeated measure. Physiological data for one participant were not recorded prior to MAST onset and therefore unavailable. Drug and MAST effects on subjective stress were assessed in a MAST (stress and control) × drug (MPH and PLC) model using univariate ANOVAs. Data for seven participants for the subjective measures were missing. The amount of food (weighted in gram) consumed, and measures of feeling hungry (%VAS line length), and feeling like eating something tasty (%VAS line length) during stage 3 (outcome devaluation) were compared between conditions using a univariate ANOVA with MAST (stress and control) and drug (PLC and MPH) as between-subject factors. For learned responses during the SOA test, a repeated measures ANOVA was performed with MAST (stress and control ) and drug (MPH and PLC) as between-subject factors and value (valuable and devalued) as repeated measure. Only significant ANOVAs were followed up by post-hoc tests. All data except fMRI data were analysed using IBM SPSS statistics 24.

## Results

### Physiological and subjective stress

#### Physiological stress

**Figure 2.**
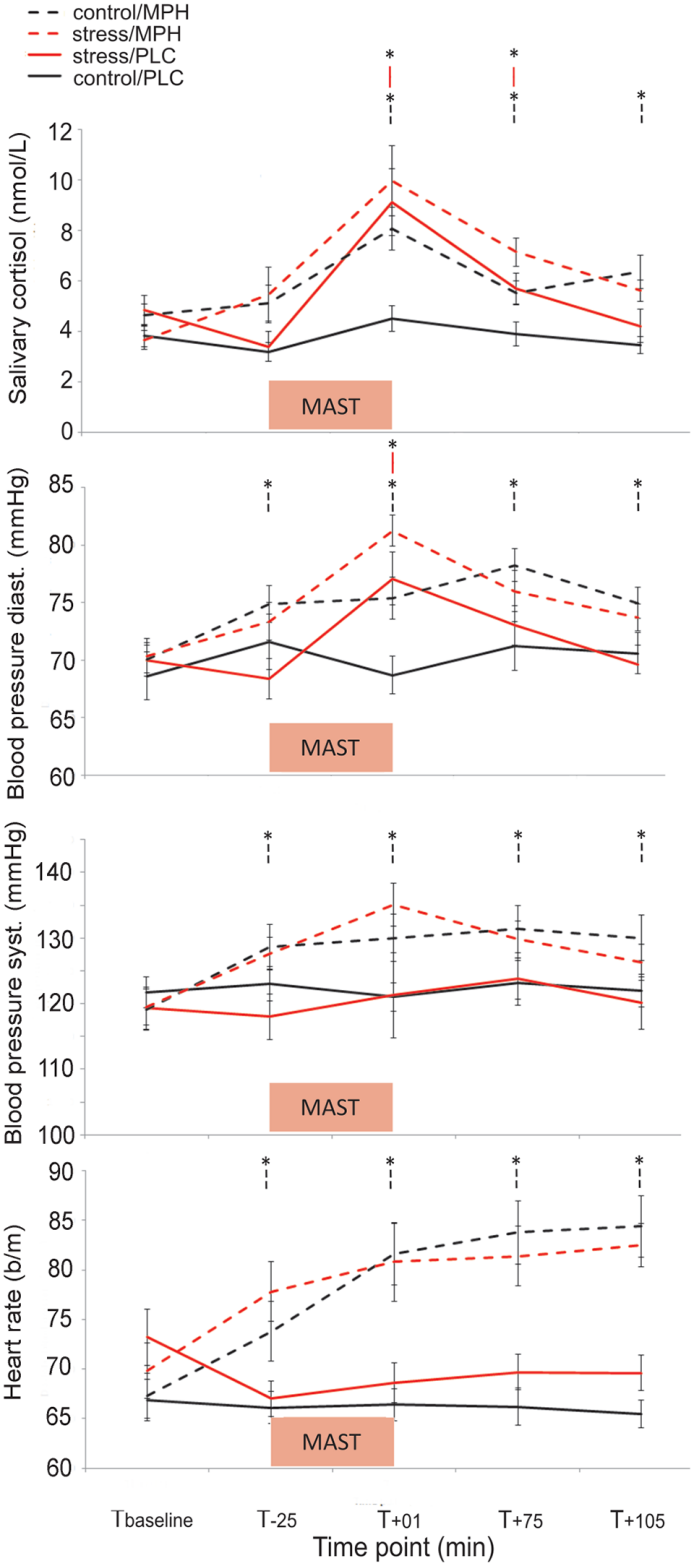
Effectiveness of stress induction. Data show untransformed means (±SE) of salivary cortisol (nmol/L), diastolic- and systolic blood pressure (mmHg), and heart rate (b/min) over time (*T*_baseline_, *T*_−25_, *T*_+01_, *T*_+75_, *T*_+105_). Both acute stress (red lines) and MPH (dashed lines) increased cortisol levels and diastolic blood pressure, but no drug-by-MAST interactions were observed. MPH additionally increased systolic blood pressure and heart rate, while stress did not. Comparisons are simple effects following significant MAST-by-time and drug-by-time interactions. *p < 0.05 compared with control/PLC.

For statistical details of all simple effects see Figure 2 and Table 1. Elevated cortisol levels were observed following the MAST in the stress relative to the control condition until 75 min after the stress induction (MAST × time: *F*(2.898,255.003) = 3.26, *p* = 0.023 η^2^_*p*_ = 0.036). Drug did not change the effect of MAST on cortisol levels over time or across all time points (drug × MAST × time: *F*(2.898,255.003) = 0.714, *p* = 0.540; drug × MAST: *F*(1,88) = 3.46, *p* = 0.066). Elevated cortisol levels were observed following MPH administration until 195 min after (i.e. 105 min after stress induction) compared with PLC (drug × time: *F*(2.898,255.003) = 3.18, *p* = 0.026, η^2^_*p*_ = 0.035). Finally, sex did not modulate any of the effects of drug or MAST or their interaction (all *Fs*(2,64) < 0.40, *p* > 0.671).

**Table 1.** Inferential statistics of MAST and drug per time point.

	MAST				Drug			
	*F*	*df*	*p*	η^2^_*p*_	*F*	*df*	*p*	η^2^_*p*_
Cortisol								
*T*_base_	0.05	1,90	0.816	–	0.01	1,90	0.972	–
*T*_−25_	0.03	1,91	0.854	–	7.50	1,91	0.007*	0.076
*T*_+01_	7.96	1,91	0.006*	0.080	8.10	1,91	0.005*	0.082
*T*_+75_	10.69	1,91	0.002*	0.105	12.84	1,91	<0.001*	0.124
*T*_+105_	0.11	1,91	0.736	–	25.39	1,91	<0.001*	0.218
Blood pressure diastolic								
*T*_base_	0.26	1,91	0.611	–	0.37	1,91	0.543	–
*T*_−25_	1.366	1,90	0.246	–	4.67	1,90	0.033*	0.049
T_+01_	15.24	1,91	0.001*	0.143	8.77	1,91	0.004*	0.088
*T*_+75_	<0.01	1,91	0.954	–	8.05	1,91	0.006*	0.081
*T*_+105_	0.39	1,91	0.532	–	7.98	1,91	0.006*	0.081
Blood pressure systolic								
*T*_base_	0.13	1,91	0.724	–	0.19	1,91	0.661	–
*T_−_*_25_	0.75	1,90	0.390	–	6.08	1,90	0.016*	0.063
*T*_+01_	0.57	1,91	0.451	–	8.00	1,91	0.006*	0.081
*T*_+75_	<0.01	1,91	0.945	–	5.01	1,91	0.028*	0.052
*T*_+105_	0.63	1,91	0.428	–	4.73	1,91	0.032*	0.049
Heart rate								
*T*_base_	2.93	1,91	0.090	–	0.25	1,91	0.620	–
*T_−_*_25_	1.09	1,90	0.299	–	14.18	1,90	<0.001*	0.136
*T*_+01_	0.12	1,91	0.735	–	23.23	1,91	<0.001*	0.203
*T*_+75_	0.10	1,91	0.755	–	31.73	1,91	<0.001*	0.259
T_+105_	0.30	1,91	0.583	–	50.34	1,91	<0.001*	0.356

* *p* < 0.05.

DBP was elevated immediately following the MAST, but not following the control condition (MAST × time: *F*(3.522,309.931) = 9.24, *p* < 0.001, η^2^_*p*_ = 0.095; see Figure 2 and Table 1), irrespective of drug (drug × MAST × time: *F*(3.522,309.931) = 0.81, *p* = 0.506). Drug did not affect DBP changes over time (drug × time: *F*(3.522,309.931) = 2.04, *p* = 0.097), but MPH did increase DBP values after administration (drug: *F*(1,88) = 8.26, *p* = 0.005, η^2^_*p*_ = 0.086).

Stress did not affect SBP over time (MAST × time: *F*(2.951,259.730) = 1.39, *p* = 0.248) or across time points (MAST: *F*(1,88) = 0.14, *p* = 0.707). Drug did not modulate the effect of stress on SBP over time (drug × MAST × time: *F*(2.951,259.730) = 0.721, *p* = 0.538) or across time (drug × MAST: *F*(1,88) = 0.11, *p* = 0.746). SBP was higher on all time points after MPH administration compared with placebo, but not before MPH (drug × time: *F*(2.951,259.730) = 5.90, *p* = 0.001, η^2^_*p*_ = 0.063).

Stress did not affect HR over time (MAST × time: (*F*(3.138,276.108) = 0.87, *p* = 0.461), independent of drug (drug × MAST × time: *F*(3.138,276.108) = 1.40, *p* = 0.243) nor across all time points (MAST: *F*(1,88) = 0.75, *p* = 0.390), independent of drug (MAST × drug: *F*(1,88) = 0.55, *p* = 0.461). HR was higher on all time points after MPH compared with placebo, but not before drug administration (drug × time: *F*(3.138,276.108) = 19.98, *p* < 0.001, η^2^_*p*_ = 0.185).

All in all, both the stress and MPH increased cortisol levels and diastolic blood pressure significantly, but MPH did not modulate the effect of stress. MPH also increased systolic blood pressure and heart rate, while stress did not.

#### Subjective stress

Participants reported increased feelings of stress (*F*(1,84) = 162.16, *p* < 0.001, η^2^_*p*_ = 0.659), pain (*F*(1,84) = 356.13, *p* < 0.001, η^2^_*p*_ = 0.809) and unpleasantness (*F*(1,84) = 107.79, *p* < 0.001, η^2^_*p*_ = 0.562) post-MAST, compared with the control condition, irrespective of drug (drug × MAST: all *Fs* < 2.61, *p* > 0.110). MPH reduced feelings of pain (*F*(1,84) = 4.58, *p* = 0.035, η^2^_*p*_ = 0.052), but did not affect feelings of stress or unpleasantness (*F*(1,84) < 0.03, *p* = 0.954 and *F*(1,84) = 3.59, *p* = 0.061, respectively).

### Goal-directed behaviour

#### S–R–O Learning

As expected, participants in all conditions learned the S–R–O associations as demonstrated by increased performance over blocks for the correct (rewarded) button press and reaching asymptotic levels. Instrumental learning increased over the course of the eight learning phase blocks (Block: *F*(3.763,334.882) = 111.76, *p* < 0.001, η^2^_*p*_ = 0.557; See Supplemental Material (Figure S1)). No drug-by-MAST interaction on instrumental learning over blocks was observed (block × drug × MAST: *F*(3.763,334.882) = 0.90, *p* = 0.462). Correct button presses did not differ over blocks between participants in the stress versus control condition (block × MAST: *F*(3.763,334.882) = 1.34, *p* = 0.257), similar to participants in both drug conditions (block × drug: *F*(3.763,334.882) = 0.87, *p* = 0.477). No drug-by-MAST interaction was observed when collapsing performance across blocks (MAST × drug: *F*(1,89) = 0.52, *p* = 0.473).

#### S–R–O *reminder*

Groups did not differ on the percentage of correct responses during the reminder phase (see Supplemental Material (Figure S1)). The drug-by-MAST interaction was not significant (*F*(1,88) = 0.08, *p* = 0.773), nor were the main effects of MAST (*F*(1,88) = 1.17, *p* = 0.283) or drug (*F*(1,88) = 0.07, *p* = 0.797), indicating that all groups performed similarly during this phase of the task.

#### Devaluation

The amount of food consumed during the devaluation procedure (in grams) did not differ between groups (all main and interaction effects involving drug and MAST: *F*s(1,89) < 1.34, all *p*s > 0.251). Moreover, no differences in the feeling of being hungry were observed between groups (drug × MAST: *F*(1,85) = 0.63, *p* = 0.431), drug: *F*(1,85) = 0.03, *p* = 0.875, MAST: *F*(1,85) = 0.23, *p* = 0.636).

A drug-by-MAST interaction was observed for the feeling of ‘wanting to eat something’ (*F*(1,84) = 4.01, *p* = 0.048, η^2^_*p*_ = 0.046). Follow-up analysis revealed that while no drug effect was observed in the control condition (*F*(1,44) = 0.13, *p* = 0.726), in the stress condition, stressed participants who received MPH felt more like eating something than stressed participants who received PLC (*F*(1,40) = 6.10, *p* = 0.018, η^2^_*p*_ = 0.132).

#### Slips-of-action test

**Figure 3.**
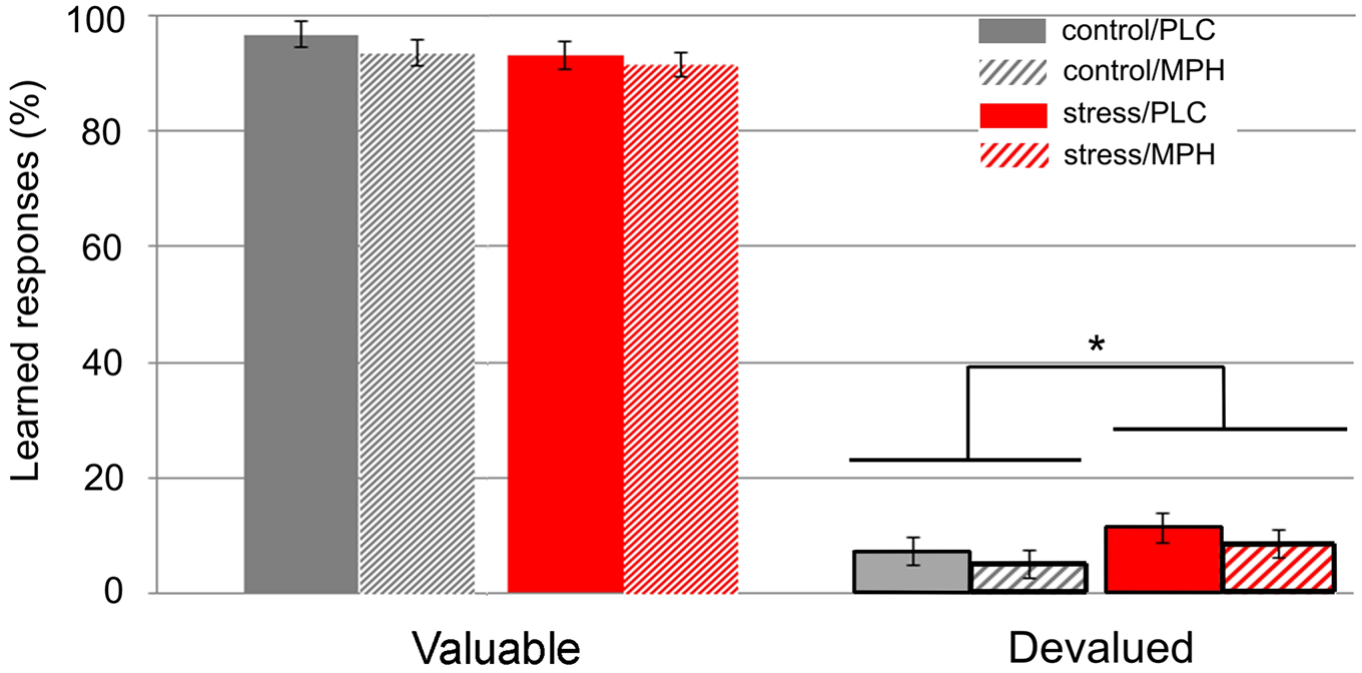
Performance on the slips-of-action task. Stress increased learned responses (%) to devalued, but not to valuable outcomes independent of drug. Note that learned responses to devalued rewards indicate reduced goal-directed behaviour. *p < 0.05.

MPH did not modulate the effect of MAST on learned responses for valued versus devalued outcomes (value × MAST × drug: *F*(1,89) = 0.03, *p* = 0.873). The differential effect of stress on learned responses for valuable versus devalued outcomes approached significance (value × MAST: *F*(1,89) = 3.91, *p* = 0.051, η^2^_*p*_ = 0.042). If stress reduces goal-directed behaviour, then these effects should be most pronounced for learned responses to devalued outcomes. Indeed, exploratory simple effects analyses revealed that participants in the stress condition exhibited an increased tendency to provide learned responses for devalued outcomes compared with controls (*F*(1,91) = 5.45, *p* = 0.022 η^2^_*p*_ = 0.056), while stress versus control participants did not differ in learned responses for valuable outcomes (*F*(1,91) = 1.83, *p* = 0.180). MPH did not affect learned responses for valuable versus devalued outcomes (value × drug: *F*(1,89) = 0.15, *p* = 0.700; Figure 3).

### Imaging results

#### Task related network: Valuable_*correct*_ – Devalued_*learned-response*_

To explore the network of brain areas involved in goal-directed behaviour (i.e. positive or negative activity difference for Valuable_correct_ – Devalued_learned-response_), a second level ANOVA was performed with these first level contrasts as input. Main effects of MAST, drug, their interaction, and the variance not explained by the main or interaction effects were modelled. Brain areas in sensorimotor and executive control networks were identified, including bilateral frontal pole/middle frontal gyrus, anterior cingulate, primary motor cortex, bilateral lateral occipital cortex, precuneous, insula, cerebellum and putamen, which were all consistent with previous work (Watson et al., 2018). See Supplemental Material (Figure S2 and Table S1).

#### Drug-dependent effects of stress on activation in regions implicated in goal-directed control

**Figure 4.**
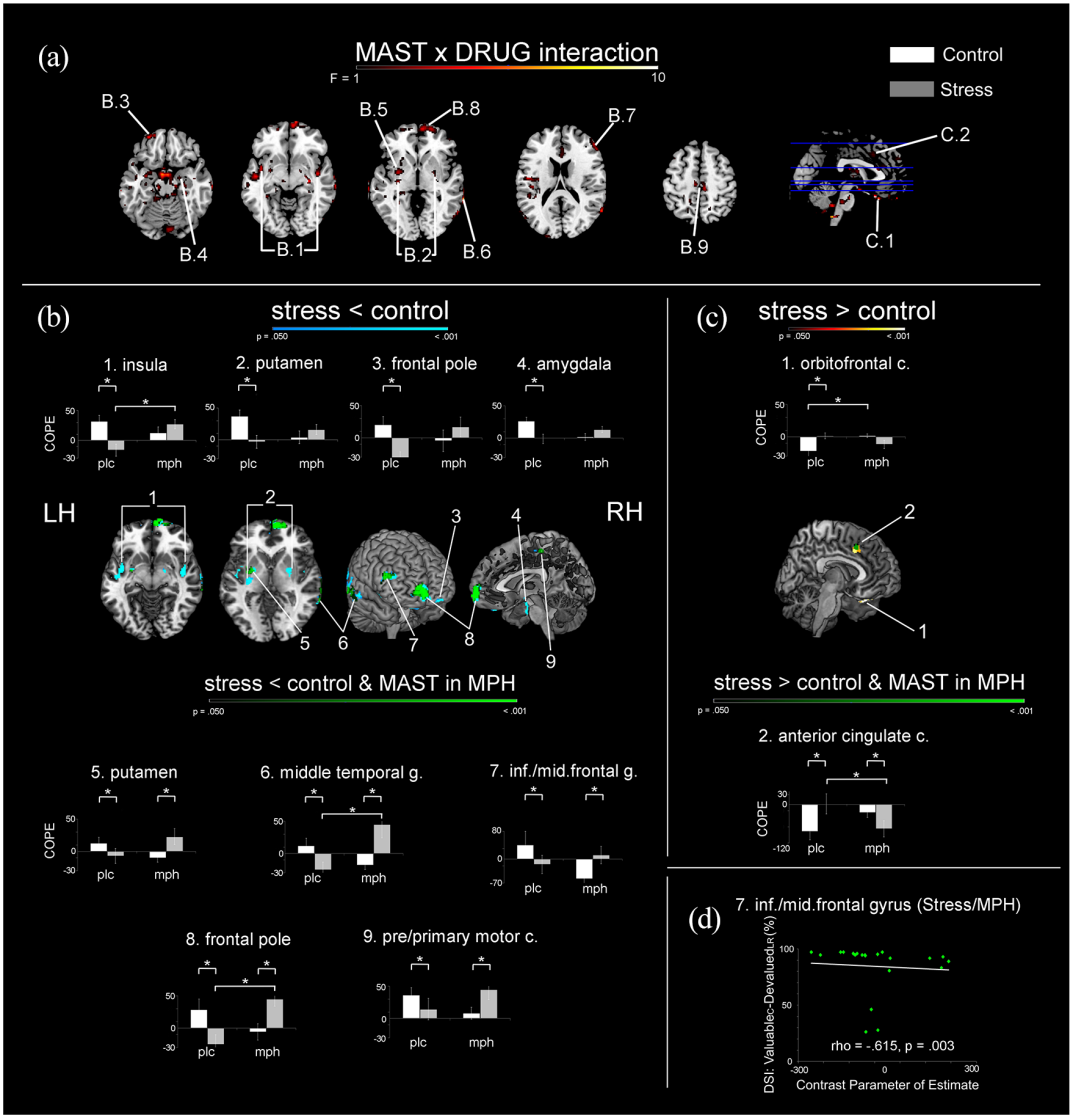
Drug-by-MAST interactions for brain activation related to goal-directed behaviour. Panel (a) Clusters of activation showing a significant drug-by-MAST interaction. Panel (b) Stress-induced reductions for *positive* Valuable_correct_ – Devalued_learned-responses_ activation differences. Significant clusters in blue indicate reduced *positive* activation difference only in participants who received placebo; green colours indicate the conjunction with the effect of stress after MPH administration. Panel (c) Stress-induced diminished *negative* difference following Valuable_correct_ – Devalued_learned-response_ in participants who received PLC (hot colours) and the conjunction with effects of stress after MPH (green). All contrasts are thresholded at *p* < 0.05, FWE, TFCE, minimal cluster size 25 voxels. Bar graphs display extracted cluster beta values ( $\bar{x}$ ± SE). Panel (d) Negative correlation between the devaluation sensitivity index (DSI: %Valuable_correct_ − %Devalued_learned-response_) and the extracted beta values for the inferior/middle frontal gyrus of stressed participants who received MPH, which indicates that a larger positive activation difference between Valuable_correct_ and Devalued_learned-response_ is associated with less goal-directed behaviour. COPE: Contrast parameter of estimate.

The analysis of the MAST-by-drug interaction for the Valuable_correct_ − Devalued_learned-response_ contrast revealed several significant clusters (Figure 4 panel (a), Table 2). The subsequent post-hoc analyses per level of the factor drug (PLC and MPH) revealed nine clusters in which stress *reduced the positive activation difference* (i.e. reduced Valuable_correct_ > Devalued_learned-response_; Figure 4 panel (b)). In two clusters, stress *reduced the negative activation difference* (i.e. reduced Valuable_correct_ < Devalued_learned-reponse_; Figure 4 panel (c)).

**Table 2.** Drug dependent effects of stress on brain activation.

Area number Figure 4	Brain area	Lat.	BA	Cluster size (voxels)	Peak *p*-value (FWE corrected)	Peak coordinates (MNI)		
						*X*	*Y*	*Z*
**B**	**Stress reduced activation after PLC**							
1	Insula	L	48	207	<0.001	*−*42	*−*4	*−*6
		R	48	81	<0.001	42	*−*8	*−*14
2	Putamen	L	48	93	<0.001	*−*30	*−*18	2
		R	35	53	<0.001	33	61	36
3	Frontal pole	L	11	39	0.002	*−*24	52	*−*16
4	Amygdala	R		50	<0.001	20	*−*8	*−*20
	**Stress reduced activation after PLC and affected activation after MPH**							
5	Putamen	L	34	88	0.002	*−*28	*−*4	*−*8
6	Middle temporal gyrus	R	21/37	85	0.005	68	*−*48	*−*4
7	Inferior/Middle frontal gyrus	R	45	62	0.002	54	36	20
8	Frontal pole	R	10	274	<0.001	8	64	*−*2
9	Pre/primary motor cortex	R	4	38	0.002	8	*−*28	54
**C**	**Stress increased activation after PLC**							
1	Orbitofrontal cortex	L	11	45	<0.001	*−*20	18	*−*26
	**Stress increased activation after PLC and affected activation after MPH**							
2	Anterior cingulate cortex	R	32	39	0.018	4	22	38

Clusters of activation showing MPH-dependent activation differences between stress and control. Section B lists clusters in which stress reduced brain activation associated with goal-directed behaviour (Valuable_correct_ − Devalued_learned-response_), both after PLC administration only and in conjunction with effects of stress following MPH. Section C lists clusters in which stress increased activation associated with goal-directed behaviour, both after PLC only and in conjunction with effects of stress after MPH administration.

L: left; R: right; Lat.: laterality; BA: Brodmann area; FWE: familywise error; MNI: Montreal Neurological Institute; PLC: placebo; MPH: methylphenidate

With regard to the positive activation difference, in 4/9 clusters (bilateral insula, bilateral putamen, left frontal pole and right amygdala; Figure 4 panel (b), top row) stress reduced brain activation associated with goal-directed behaviour only in participants who received placebo. In these regions, no effect of stress on brain activation associated with goal-directed behaviour was observed in participants who received MPH. For the remaining five clusters (left putamen, right middle temporal gyrus, right inferior/middle frontal gyrus, frontal pole and right pre-/primary motor cortex; Figure 4 panel (b), bottom row), stress increased brain activation associated with goal-directed behaviour in participants who received MPH.

Importantly, using *t*-contrasts, we observed that MPH reversed the effect of acute stress (relative to the effect of stress observed in the PLC group) in right middle temporal gyrus and frontal pole; in these regions, the positive activation difference for Valuable_correct_ versus Devalued_learned-reponse_ trials was greater for participants in the MPH/stress condition compared with participants in the PLC/stress condition, and participants in the MPH/stress condition did not statistically differ from participants in the PLC/control condition. We observed a similar pattern in the bilateral insula, but in contrast, here MPH/stress and MPH/control participants did not statistically differ from each other.

With regard to the two *negative activation difference clusters*, we observed that stress was associated with a reduction in the activation difference between Valuable_correct_ and Devalued_learned-response_ in orbitofrontal cortex and anterior cingulate cortex. In the right anterior cingulate, MPH successfully reversed the effect of stress on goal-directed behaviour associated activation: the negative activation difference for Valuable_correct_ versus Devalued_learned-reponse_ trials was smaller for PLC/stress than for MPH/stress participants, and PLC/control and MPH/stress participants did not differ from each other.

For exploratory purposes, a full list of all peak voxels that were not a priori associated with instrumental behaviour (goal-directed or habitual), but that did show significant effects of MAST (in PLC and/or MPH groups) is presented in Supplemental Material (Table S2). The effect of MPH in control (i.e. no-stress) participants is reported in Supplemental Material (Figure S3 and Table S3).

### Associations between brain activity and goal-directed *behaviour*

Correlations between brain activation associated with goal-directed behaviour (contrast Valuable_correct_ − Devalued_learned-responses_) and the percentage of learned responses for valuable−devalued trials, which is considered to be a measure of sensitivity to outcome devaluation (DSI; Watson et al., 2018), were performed per group, per significant cluster. Only the negative correlation for the inferior/middle frontal gyrus in stressed participants who received MPH remained significant after correcting for multiple comparisons (ρ = −0.615, *p* = 0.003; see Figure 4 panel (d)). All other correlations were not significant (*p*’s > 0.006)

## Discussion

We aimed to investigate how catecholamines contribute to stress-induced changes in goal-directed behaviour and associated brain activation, using a combination of experimental stress induction and MPH administration. The MAST successfully increased subjective ratings and objective (i.e. salivary cortisol levels and vital signs) measures of stress, and MPH increased salivary cortisol levels, vital signs and decreased feelings of pain, suggesting that both manipulations were successful.

Acute stress seemed to increase the tendency of participants to exert actions (button presses) associated with obtaining a devalued outcome (i.e. ‘learned responses’), representing a reduction in goal-directed behaviour. Performance on trials involving non-devalued outcomes, however, were similar across all groups, suggesting that acute stress was not associated with more general impairments in instrumental behaviour. The observation that stress tended to reduce goal-directed behaviour corroborates previously observed effects of stress on instrumental behaviour (e.g. Quaedflieg et al., 2019; Smeets et al., 2019, for review see Schwabe and Wolf, 2011), and action-outcome learning specifically (e.g. Otto et al., 2013).

Contrasting trials that presumably involve more (learned responses for *non-devalued* outcomes) and less (learned responses for *devalued* outcomes) goal-directed behaviour, we observed activity differences in, among others, insula, putamen, anterior cingulate and lateral prefrontal cortex, consistent with previous work (e.g. de Wit et al., 2012; Watson et al., 2018). Activation of the putamen and insula has been consistently observed in studies using outcome devaluation paradigms (Balleine and Dickinson, 2000; de Wit et al., 2012; Watson et al., 2018). The putamen has been argued to track outcome probabilities (Brovelli et al., 2011) and putamen and insula both encode aspects of reward value (Peterson and Seger, 2013; Smith et al., 2009). As part of the salience network, the insula moreover plays a role in assigning incentive value to outcomes based on saliency (Balleine and Dickinson, 2000), facilitates action selection (Oldham et al., 2018) and has previously been implicated in habitual behaviour control (Watson et al., 2018). These reports are in line with our observed finding of greater activation in these regions during learned responses for valuable compared with devalued outcomes.

Acute stress was associated with widespread reductions in activation differences in regions associated with goal-directed behaviour, including bilateral putamen, insula, inferior/middle frontal gyrus and right amygdala. It is thought that less successful goal-directed behaviour is associated with relatively high putamen activation for devalued rewards (Graybiel and Grafton, 2015). Reduced activity in OFC for actions with greater outcome value is also consistent with stress-induced reductions in reward-related medial PFC responses (Ossewaarde et al., 2011), and changes in OFC activation during simultaneous modulation of glucocorticoid and noradrenaline systems (Schwabe et al., 2012). Differences in stress-induced reductions in OFC and ACC activation associated with goal-directed behaviour may contribute to reduced differentiation in judgement of reward/outcome (Graybiel and Grafton, 2015; Quaedflieg et al., 2019), a reduced ability to inhibit learned responses (Verbruggen and Logan, 2008) and impaired response conflict resolution (Botvinick et al., 2009) under stress, which may thus result in the use of ‘habitual’ strategies.

Importantly, although MPH did not modulate task performance, pre-treatment with MPH did prevent a stress-induced shift in brain activation associated with goal-directed behaviour. In the insula, middle temporal gyrus, frontal pole and anterior cingulate cortex, stressed participants who received MPH displayed similar activation levels compared with non-stressed participants who received PLC (while activation levels differed from participants in the stress/PLC and no-stress/MPH conditions). It is well known that acute stress increases dopamine release in cortical and striatal regions (Hernaus et al., 2015; Nagano-Saito et al., 2013; Vaessen et al., 2015). Moreover, L-DOPA administration modulates model-based control of behaviour (Wunderlich et al., 2012), and administration of dopaminergic agonists and antagonists have been linked to selective changes in sensitivity to positive and negative outcomes (Frank et al., 2004). On the other hand, MPH effects on brain activation may also be partly noradrenergic, since MPH has a higher binding potential for noradrenaline transporters compared with the dopamine transporter (Hannestad et al., 2010; Volkow et al., 2002). Moreover, noradrenaline increases seem to normalise dorsal striatum-mPFC connectivity following rewards preceded by cues, thereby enhancing the discrimination between reward and non-rewarded cues in ADHD (Furukawa et al., 2020). Finally, the negative correlation between the DSI as a measure of goal-directed tendencies and the activation difference between Valuable_correct_ and Devalued_learned-response_ in the inferior/middle frontal gyrus in stressed participants who received MPH may be indicative of MPH contributing to goal-directed behaviour under stress. Taken together, these results suggest that both MPH-induced dopamine and noradrenaline increases may have contributed to normalisation of brain activation associated with goal-directed behaviour under stress.

One potential mechanism-of-action of the observed MPH effects under stress may involve modulation of signal-to-noise ratio in cortical networks. Dopamine and noradrenaline jointly control the signal-to-noise ratio of neural activity in frontal cortical networks, associated with optimal cognitive performance (e.g. Arnsten, 2009a; Vijayraghavan et al., 2007), and acute stress reduces PFC signal-to-noise ratio (Arnsten, 2009b), which, in the current study, could be reflected by reduced activation differences between learned responses for valuable and devalued outcomes under stress. The administration of MPH, via changes in phasic dopamine/noradrenaline firing (Evers et al., 2017), may have thus prevented stress-induced changes in neuronal signal-to-noise ratio. This interpretation would also align with interactions between MPH- and stress-induced frontal cortex dopamine release in rodents (Marsteller et al., 2002).

The observed pattern in the four treatment groups seemed to follow a quadratic trend in the middle temporal gyrus, frontal pole and anterior cingulate cortex. Here, the pattern of brain activation associated with goal-directed behaviour was similar for participants in no-stress/PLC and stress/MPH conditions on the one hand, and for participants in the stress/PLC and no-stress/MPH conditions on the other hand. The observation of an (inverted) U curve has been well-established in the context of cognitive performance and dopamine function (Arnsten, 2009b; Arnsten and Goldman-Rakic, 1998; Cools and D’Esposito, 2011; Goldman-Rakic et al., 2000). To our knowledge, this is the first study to demonstrate in humans how acute stress and stimulants might jointly facilitate shifts in the position along this U curve, via changes in putative dopaminergic mechanisms. Future studies may aim to further explore how varying levels of dopamine agonism (via acute stress or psychopharmacological agents) may induce shifts along this U curve. This will also contribute to a better understanding of interindividual differences in dopamine levels, and their association with cognitive processes and associated brain activation under stress.

Some limitations of the current study should be acknowledged. First, our conclusions are derived from a relatively small behavioural effect. The low number of SOA in all conditions may signal the relative absence of habit formation and limited the sensitivity to detect stress-induced reductions in goal-directed behaviour, and its potential reversal by MPH on a behavioural level. Similarly, the low number of SOA may have affected the signal-to-noise ratio of the fMRI measurement. However, the number of trials associated with a habitual response is similar to that in Watson et al. (2018) who presented comparable results. In addition, Steele et al. (2016) have shown that the 4–10 trials with a sample size of 20 participants produces reliable signals in an error processing task. Another limitation concerns potential boundary conditions associated with stress effects on goal-directed behaviour. One such condition is that stress-induced changes in instrumental behaviour may be limited to participants characterised by low working memory capacity (Quaedflieg et al., 2019). Our sample consisted mostly of academic students who are expected to have relatively high working memory capacity, which may protect against performance impairments under stress. Next, hormonal contraceptives and variation in menstrual cycle may have affected the stress response (Kirschbaum et al., 1999). As data from the current experiment unfortunately do not allow for a sufficiently powered analysis of effects of the menstrual cycle or hormonal contraceptives, future studies could systematically examine potential menstrual cycle phase effects.

The current findings may be relevant to our understanding of stress-associated relapse behaviour in addiction (Sinha, 2001); stress may reduce goal-directed behaviour and thus could prompt reliance on old habits, such as drug-taking behaviour, in individuals suffering from addiction. Our observation that dopamine and noradrenaline contribute to changes in brain activation associated with goal-directed behaviour under stress aligns well with the role of dopamine, particularly in the dorsal striatum, in habit formation (Belin et al., 2013; Gasbarri et al., 2014; Nelson and Killcross, 2013), which are thought to be D2 receptor-mediated (Kwak et al., 2014; Volkow et al., 2006, 2013). Administration of MPH to individuals suffering from addiction may enhance cortico-striatal dopamine function, ultimately enhancing frontal cortex based goal-directed behaviour. This idea is supported by the observation that MPH administration to cocaine-dependent individuals normalises anterior cingulate activation and increases inhibitory control (Li et al., 2010). The use of stimulants in these populations, however, should be closely monitored given that stimulants also increase the motivation to gamble (Zack and Poulos, 2004), and have been reported to increase striatal dopamine release in pathological gamblers (Boileau et al., 2014).

To conclude, stress-induced reductions in brain activation associated with goal-directed behaviour may involve diminished differentiation between valuable and devalued rewards. These effects may be driven by both changes in expected value associated with OFC and ACC activation, and in action selection associated with activation of the putamen and insula. MPH seemed to reverse this stress-induced reduction in activation differences in the insula, middle temporal gyrus, frontal pole and ACC implying that dopamine and noradrenaline may drive stress-induced changes in representations of reward value. However, MPH did not impact goal-directed behaviour. Future studies could be conducted to examine the many boundary conditions related to the stress-induced shift in goal-directed behaviour (e.g. working memory capacity, oral contraceptives, baseline dopamine levels) and further disentangle the association between catecholamine function, stress and brain activation underlying goal-directed behaviour.

## Supplemental Material

sj-docx-1-jop-10.1177_02698811211044679 – Supplemental material for Dopaminergic and noradrenergic modulation of stress-induced alterations in brain activation associated with goal-directed behaviour Click here for additional data file. Supplemental material, sj-docx-1-jop-10.1177_02698811211044679 for Dopaminergic and noradrenergic modulation of stress-induced alterations in brain activation associated with goal-directed behaviour by Peter van Ruitenbeek, Conny WEM Quaedflieg, Dennis Hernaus, Bart Hartogsveld and Tom Smeets in Journal of Psychopharmacology
