# Supplementary material for: Dopaminergic and noradrenergic modulation of stress-induced alterations in brain activation associated with goal-directed behaviour
Source: J Psychopharmacol. 2021 Sep 14;35(12):1449–63. doi: 10.1177/02698811211044679 (PMC8652367; doi:10.1177/02698811211044679)
Supplement: sj-docx-1-jop-10.1177_02698811211044679 – Supplemental material for Dopaminergic and noradrenergic modulation of stress-induced alterations in brain activation associated with goal-directed behaviour [file sj-docx-1-jop-10.1177_02698811211044679.docx]

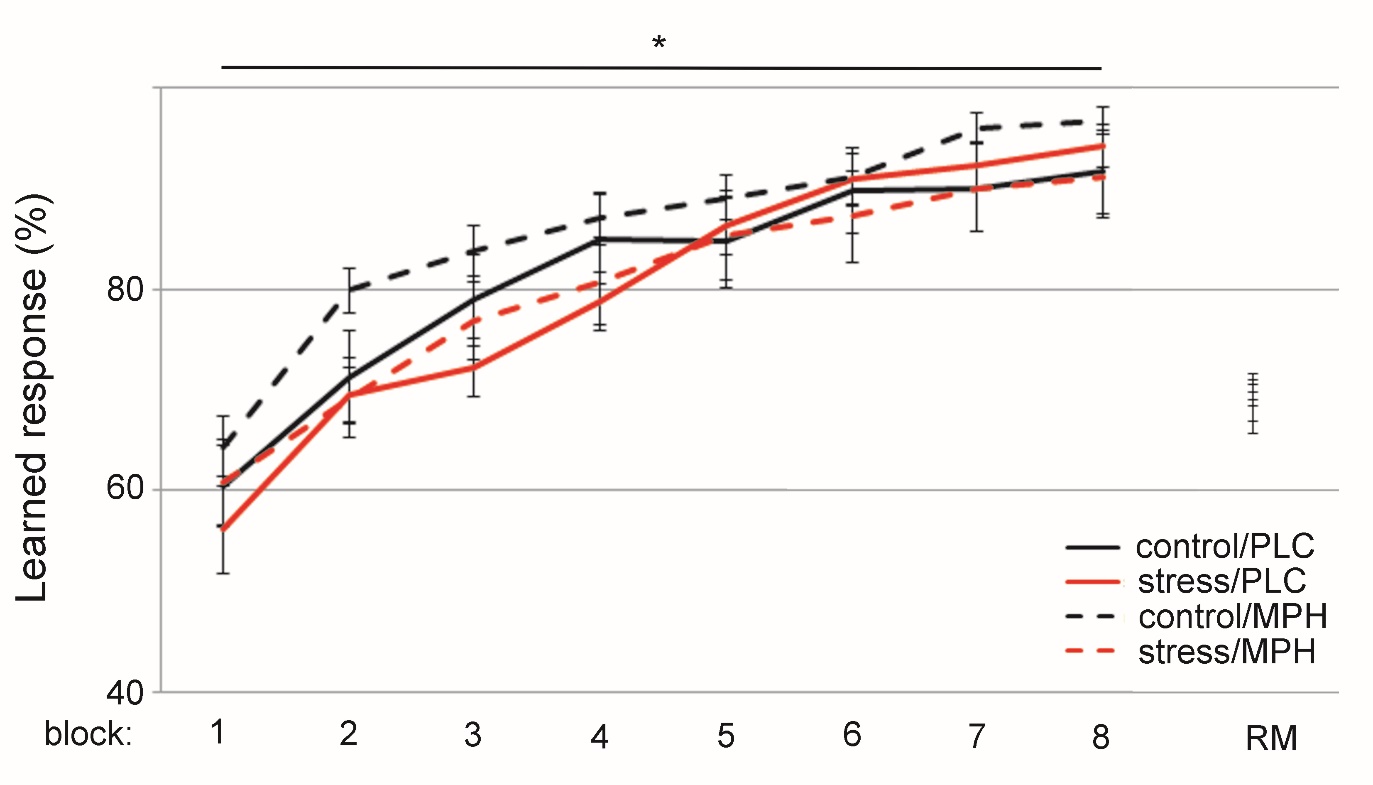


**Figure S1:**  **Performance on the instrumental learning task during training and reminder phase**

Performance on the instrumental learning task significantly increased over blocks during the learning phase (**F*(3.763,334.882) = 111.76, *p* < .001, *η*_p_^2^ = .557), reaching high level of performance during the final blocks. No group differences were observed across and between time points, nor during the reminder phase.


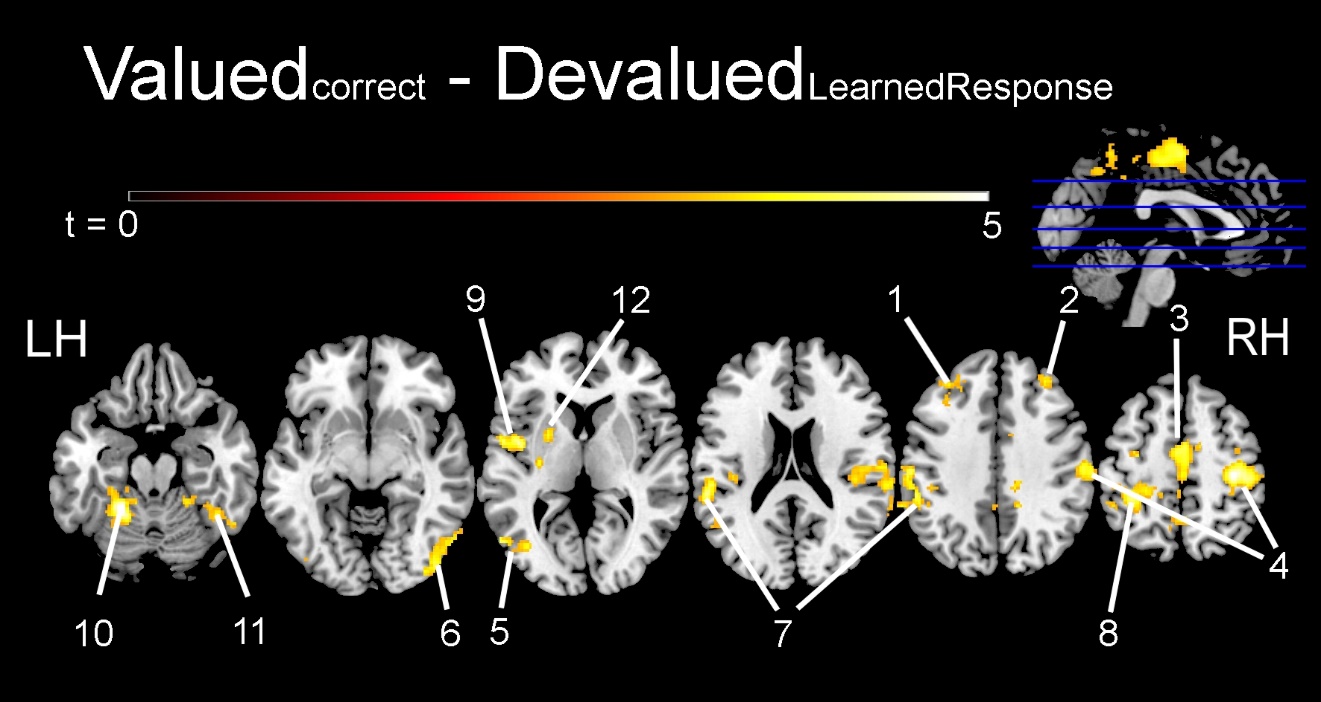


**Figure S2:** **Brain activation associated with making a goal-directed response.**

The contrast between brain activation associated with making a learned response on valuable trails (Valuable_correct_) and learned responses (i.e. incorrect responses) to obtain devalued rewards (Devalued_learned-response_), which we considered a measure of brain activation associated with goal-directed behaviour. Brain areas in among others sensorimotor and executive control networks were identified. The numbers correspond to the numbers in table S1 that provides descriptive statistics of the clusters.


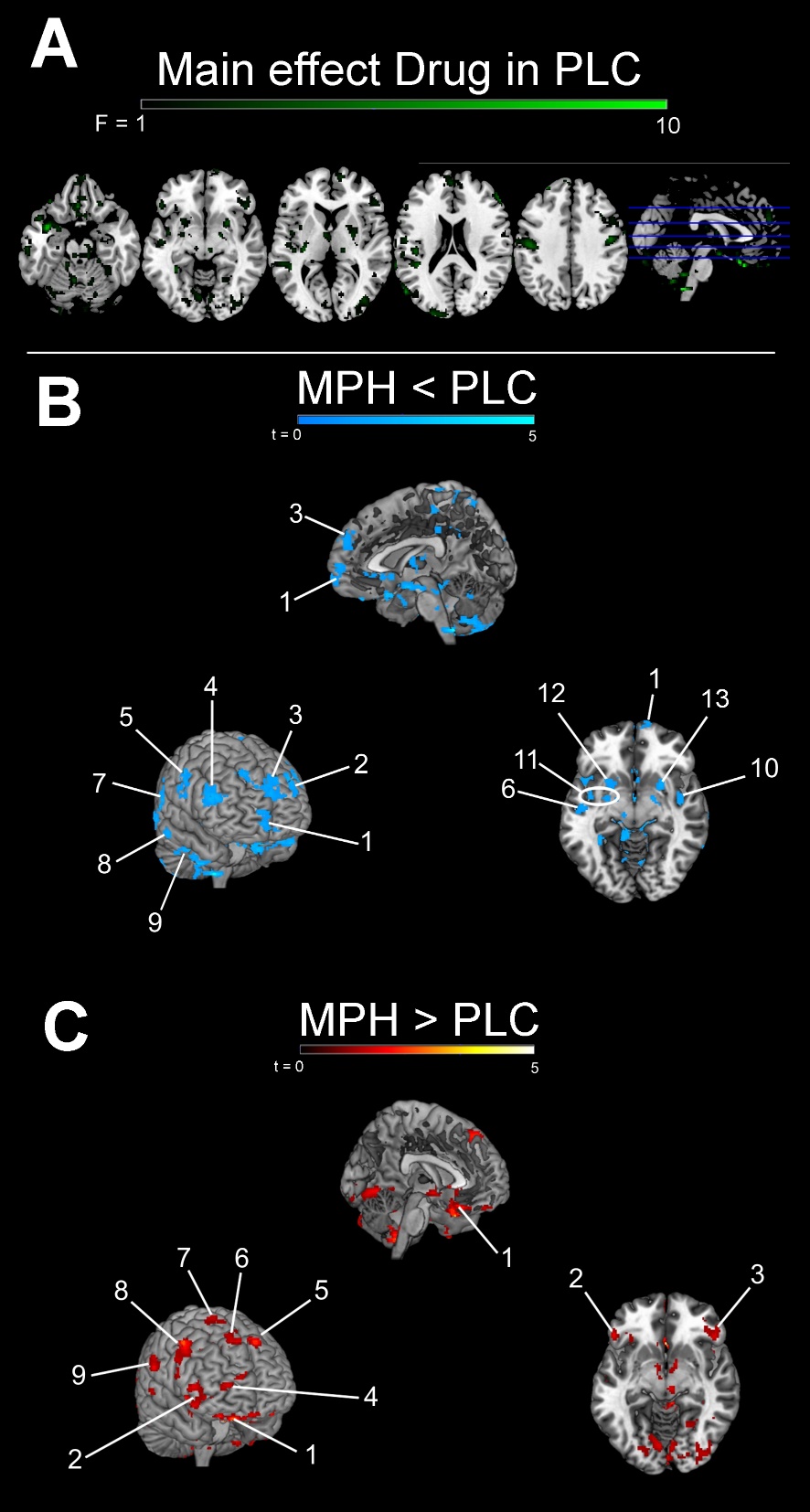


**Figure S3:** **Effects of MPH on brain activation associated with goal-directed behaviour in the MAST control condition.** Panel A: Bidirectional F-statistics for the effects of MPH on brain activation differences between Valued_correct_ and Devalued_LearnedResponse_ trials representing goal-directed behaviour associated brain activation. Panel B: MPH-induced decreases in activation difference between task conditions. The numbers correspond to the numbers in table S3 that provides descriptive statistics of the clusters.Panel C: MPH-induced increases in the difference between task conditions.

| Area  Number  Figure S2 |  |  |  | Cluster size (voxels) | Peak p-value  (FWE corrected) | Peak coordinates (MNI) | | |
| --- | --- | --- | --- | --- | --- | --- | --- | --- |
|  | Brain area | Lat. | BA |  |  | X | Y | Z |
| **Goal-directed behaviour associated activation (Valuable_correct_ – Devalued_Learned Response_)** | | | | | | | | |
| 1 | Middle frontal gyrus | L | 9/46 | 246 | .001 | -36 | 30 | 46 |
| 2 | Frontal pole | R | 9/46 | 177 | .008 | 34 | 40 | 32 |
| 3 | Anterior cingulate | R | 24 | 685 | <.001 | 6 | 4 | 42 |
| 4 | Primary motor cortex | R | 4 | 2896 | <.001 | 34 | -24 | 56 |
| 5 | Lateral occipital cortex | L | 37 | 138 | .025 | -56 | -68 | 4 |
| 6 |  | R | 19 | 432 | <.001 | 44 | -84 | -6 |
| 7 | Inferior parietal lobule | L | 48 | 2402 | <.001 | -58 | -20 | 30 |
| 8 | Precuneous | L | 5 | 398 | <.001 | -4 | -52 | 48 |
| 9 | Insula | L | 48 | 301 | <.001 | -42 | -2 | 6 |
| 10 | Cerebellum V | L | - | 852 | <.001 | -22 | -46 | -20 |
| 11 |  | R | - | 186 | .006 | 20 | -42 | -18 |
| 12 | Putamen | L | - | 182 | .007 | -24 | 4 | 4 |

**Table S1: Brain regions displaying activation associated with goal-directed behaviour.** Brain areas showing significantly more activation on Valuable_correct_ trails compared with Devalued_learned response_ trials and corrected for effects of MAST, Drug and their interaction.

**Table S2: Drug dependent effects of stress on brain activation**

A comprehensive overview of all peak voxels within clusters of activation showing MPH-dependent activation differences between MAST-induced stress and control differences. Section A lists clusters in which stress reduced brain activation associated with goal-directed behaviour (i.e. Valuable_correct_-Devalued_learned-response_ contrast), both after placebo administration only and in conjunction with effects of stress following MPH. Section B lists clusters in which stress increased activation associated with goal-directed behaviour, both after placebo only and in conjunction with effects of stress after MPH administration. Areas of interest are presented in the main paper, not here. L = left R = right Lat. = laterality BA = Brodmann area FWE = familywise error MNI = Montreal Neurological Institute PLC = placebo MPH = methylphenidate

|  |  |  | Cluster size (voxels) | Peak p-value  (FWE corrected) | Peak coordinates (MNI) | | |
| --- | --- | --- | --- | --- | --- | --- | --- |
| Brain area | Lat. | BA |  |  | X | Y | Z |
| **A** | **Stress reduced activation after PLC** | | | | | | |
| Middle frontal gyrus | L | 46 | 33 | .041 | -42 | 32 | 42 |
| Superior parietal lobule 5Ci | L | - | 33 | .012 | -14 | -34 | 48 |
| 7A | R | 7 | 119 | .005 | 14 | -60 | 66 |
| Inferior parietal lobule PFcm | L | 41 | 191 | <.001 | -48 | -36 | 14 |
| Somatosensori cortex | R | 3 | 82 | .012 | 20 | -34 | 66 |
| Temporal pole | L | 38 | 186 | <.001 | -36 | 4 | -22 |
|  |  |  | 97 | .012 | -50 | 14 | -24 |
| Primary auditory cortex | R | 20 | 286 | <.001 | 44 | 0 | -46 |
| Entorhinal cortex | L | 35 | 106 | <.001 | -14 | -14 | -28 |
|  | R | 36 | 80 | <.001 | 28 | 4 | -28 |
| Hippocampus | L | 37 | 74 | .005 | -26 | -36 | 0 |
| Dentate gyrus | R | 20 | 49 | .005 | 30 | -22 | -10 |
| Fusiform cortex | L | 14 | 62 | .005 | -32 | -44 | -14 |
| Lateral occipital cortex | L | 39 | 116 | .002 | -54 | -70 | 20 |
|  | R | 19 | 80 | .005 | 40 | -84 | 28 |
| Cerebellum Crus I | R | - | 36 | .002 | 40 | -82 | -26 |
| Crus II | L | - | 84 | <.001 | -18 | -90 | -32 |
| VIIIb | R | - | 364 | <.001 | 24 | -46 | -52 |
| I-IV | L | 30 | 116 | <.001 | -10 | -42 | -12 |
| VI | R | - | 50 | .005 | 14 | -68 | -28 |
| VIIIa | R | - | 57 | <.001 | 6 | -68 | -52 |
| IX | R | - | 28 | .012 | 8 | -46 | -36 |
| Putamen | L | 48 | 35 | .002 | -22 | 18 | -2 |
| Thalamus | L/R | - | 47 | .012 | 0 | -6 | 10 |
| Brain stem | L | - | 169 | .012 | -10 | -36 | -48 |
|  | R | - | 42 | <.001 | 2 | -46 | -56 |
| Mamillary bodies | L | - | 90 | .002 | -6 | -10 | -16 |
| **Stress reduced activation after PLC and affected activation after MPH** | | | | | | | |
| Superior parietal lobule 7A | R | 7 | 80 | .005 | 12 | -60 | 66 |
| Inferior parietal lobule PGp | L | 39 | 28 | .002 | -50 | -70 | 32 |
| PGa | L | 19 | 27 | .021 | -38 | -70 | 32 |
| Lateral occipital cortex | R | 19 | 65 | .005 | 38 | -84 | 28 |
| Angular gyrus | R | 21 | 54 | .003 | 64 | -56 | 22 |
| Superior temporal gyrus | L | 20 | 117 | <.001 | -44 | -12 | -14 |
| Middle temporal gyrus | L | 21 | 79 | .012 | -48 | -40 | 2 |
| Inferior temporal gyrus | R | 20 | 77 | <.001 | 42 | 0 | -46 |
| Temporal pole | L | 38 | 63 | .021 | -44 | 20 | -34 |
|  | R | 38 | 103 | <.001 | 36 | 22 | -32 |
| Cerebellum crus II | L | - | 61 | .002 | -18 | -88 | -32 |
| VIIIb | R | - | 251 | <.001 | 24 | -46 | -52 |
| Parahippocampal gyrus | L | 29 | 120 | <.001 | -8 | -40 | -12 |
|  |  | 35 | 52 | <.001 | -12 | -12 | -26 |
|  | R | 36 | 53 | <.001 | 24 | -12 | -36 |
| Dentate gyrus | L | 37 | 61 | .012 | -24 | -34 | 0 |
| Hippocampus | R | 20 | 35 | .005 | 30 | -22 | -10 |
| Mamillary bodies | L | - | 73 | .003 | -6 | -6 | -16 |
| Thalamus | L | - | 33 | .012 | -2 | -6 | 10 |
| Brain stem | L | - | 122 | .012 | -10 | -36 | -48 |
| **B** | **Stress increased activation after PLC** | | | | | | |
| Occipital pole | R | 18 | 55 | <.001 | 4 | -92 | -14 |
| Cerebellum I-IV | R | - | 45 | <.001 | 4 | -42 | -26 |
| **Stress both increased activation after PLC and affected activation after MPH** | | | | | | | |
| Lingual gyrus | R | 18 | 31 | .003 | 6 | -86 | -16 |

**Table S3: MPH effects on brain activation associated with goal-directed behaviour**

Section A lists clusters in which MPH decreased activation associated with goal-directed behaviour during the MAST control condition. Section B lists clusters in which MPH increased brain activation associated with goal-directed behaviour (i.e. Valuable_correct_-Devalued_Learned-response_ contrast) in the MAST control condition.

| Area  Number  Figure S3 |  |  |  | Cluster size (voxels) | Peak p-value  (FWE corrected) | Peak coordinates (MNI) | | |
| --- | --- | --- | --- | --- | --- | --- | --- | --- |
|  | Brain area | Lat. | BA |  |  | X | Y | Z |
| **A** |  | **MPH decreased activation** | | | | | | |
|  | Subcallosal cortex | R | 25 | 45 | <.001 | 0 | 22 | -6 |
|  | Orbitofrontal cortex | R | 25 | 71 | <.001 | 12 | 16 | -22 |
|  | Medial frontal cortex | R | 11 | 73 | <.001 | 2 | 26 | -30 |
|  |  |  | 11 | 55 | <.001 | 4 | 42 | -32 |
| 1 | Frontal pole | R | 11 | 126 | <.001 | 14 | 70 | -8 |
| 2 |  |  | 46 | 87 | <.001 | -28 | 50 | 36 |
|  |  |  | 9 | 36 | <.001 | 24 | 50 | 42 |
| 3 | Superior frontal gyrus (medial) | L | 10 | 331 | <.001 | -2 | 54 | 24 |
| 4 | Middle frontal gyrus | R | 45 | 162 | <.001 | 54 | 36 | 20 |
|  |  |  | 9 | 50 | <.001 | -44 | 24 | 46 |
|  | Inferior frontal gyrus | R | 45 | 60 | <.001 | -56 | 30 | 12 |
|  | Anterior cingulate | R | 11 | 36 | <.001 | -2 | 32 | -6 |
|  | Premotor cortex | L | 4/6 | 29 | <.001 | -6 | -18 | 60 |
|  |  |  | 6 |  | <.001 | -10 | -14 | 74 |
|  | Precentral gyrus | L | 6 | 26 | <.001 | -32 | -12 | 50 |
|  | Primary motor cortex | L | 48 | 778 | <.001 | -66 | -16 | 24 |
|  |  |  | 4 | 39 | <.001 | 8 | -26 | 56 |
| 5 |  | R | 4 | 243 | <.001 | 46 | -10 | 34 |
|  |  |  | 4 | 136 | <.001 | 14 | -30 | 74 |
|  | Posterior cingulate | L | 23 | 78 | <.001 | -2 | -30 | 38 |
|  |  |  | - | 37 | <.001 | 14 | -42 | 38 |
|  | Primary somatosensori cortex | L | 5 | 78 | <.001 | -16 | -46 | 56 |
|  |  |  | 4 | 73 | <.001 | -20 | -36 | 74 |
|  | Superior parietal lobule 7A | L | 5 | 113 | <.001 | 16 | -58 | 70 |
|  | Inferior parietal lobule PGp | L | 19 | 125 | <.001 | 42 | -80 | 32 |
|  | PFcm |  | 41 | 97 | <.001 | -40 | -38 | 20 |
|  | Precuneous | L | 30 | 34 | 0.001 | -12 | -54 | 10 |
|  | Lateral occipital cortex | L | 39 | 258 | <.001 | -54 | -70 | 22 |
|  |  |  | 37 | 40 | 0.001 | 58 | -66 | 0 |
|  | Temporal fusiform cortex | L | 37 | 142 | <.001 | -34 | -44 | -8 |
|  | Supramarginal gyrus | L | 22 | 35 | <.001 | -66 | -38 | 22 |
| 6 | Superior temporal gyrus | L | 48 | 70 | <.001 | -48 | -12 | -10 |
| 7 |  | R | 22 | 64 | <.001 | 70 | -34 | 16 |
|  | Superior/Middle temporal gyrus | L | 21 | 131 | <.001 | -46 | -40 | 2 |
|  | Middle temporal gyrus | L | 21 | 48 | <.001 | -64 | -54 | 0 |
| 8 | Middle temporal gyrus | R | 21 | 44 | <.001 | 70 | -44 | -2 |
|  |  |  | 21 | 28 | 0.001 | 70 | -20 | -16 |
|  |  |  | 21 | 26 | <.001 | -60 | -2 | -32 |
| 9 |  |  | 20 | 92 | <.001 | 52 | -14 | -32 |
|  |  |  | 20 | 33 | <.001 | -50 | -20 | -34 |
|  |  |  | 20 | 26 | <.001 | 44 | -16 | -40 |
|  | Inferior temporal gyrus | R | 20 | 104 | <.001 | 46 | 8 | -46 |
|  | Temporal pole/amygdala | L | 38 | 366 | <.001 | -36 | 4 | -22 |
|  | Insula | L | 48 | 36 | <.001 | -32 | -14 | 14 |
| 10 |  |  | 48 | 29 | 0.001 | 44 | -8 | -8 |
|  | Cerebellum crus I | L | - | 39 | <.001 | 42 | -40 | -36 |
|  | crus II | L | - | 58 | <.001 | 38 | -80 | -42 |
|  | IX | L/R | - | 242 | <.001 | 0 | -56 | -46 |
| 11 | Insula/Putamen | L | - | 183 | <.001 | -42 | -2 | -10 |
| 12 | Putamen | R | - | 120 | <.001 | -24 | 12 | -6 |
| 13 |  |  | - | 97 | <.001 | 26 | 10 | -10 |
|  | Brain stem/cerebellum | R | - | 1693 | <.001 | 4 | -2 | -16 |
|  |  |  | - | 426 | <.001 | 4 | -44 | -56 |
|  | Brain stem | R | - | 129 | <.001 | 4 | -42 | -32 |
|  | Thalamus | L | - | 127 | <.001 | -4 | -6 | 6 |
| **B** |  | **MPH increased activation** | | | | | | |
|  | Subcallosal cortex | L | 11 | 27 | <.001 | -8 | 20 | -10 |
| 1 | Orbitofrontal cortex | L | 11 | 462 | <.001 | -16 | 12 | -28 |
| 2 |  |  | 38/47 | 128 | <.001 | -48 | 24 | -14 |
|  |  |  | 47 | 71 | <.001 | -34 | 32 | -20 |
|  |  | R | 47 | 44 | <.001 | 26 | 28 | -12 |
| 3 | Frontal pole | R | 47 | 269 | <.001 | 50 | 34 | -14 |
| 4 |  |  | 10 | 56 | <.001 | 34 | 56 | 4 |
|  |  |  | 10 | 25 | <.001 | 22 | 62 | 14 |
| 5 | Superior frontal gyrus | L | 8/9 | 149 | <.001 | -8 | 36 | 42 |
|  |  |  | 6 | 30 | <.001 | -24 | 4 | 58 |
| 6 |  | R | 32 | 146 | <.001 | 18 | 38 | 40 |
| 7 |  |  | 6 | 62 | <.001 | 22 | 10 | 66 |
|  |  |  | 8 | 35 | <.001 | 22 | 8 | 48 |
|  | Middle frontal gyrus | L | 44 | 68 | <.001 | -42 | 10 | 40 |
| 8 |  | R | 44 | 422 | <.001 | 48 | 8 | 26 |
|  | Inferior frontal gyrus | R | 6 | 26 | <.001 | 56 | 10 | 14 |
|  | Postcentral gyrus | R | 2 | 78 | <.001 | 66 | -22 | 22 |
|  | Secondary somatosensory cortex | L | 48 | 65 | <.001 | -42 | -24 | 14 |
|  | Inferior parietal lobule | L | 42 | 57 | <.001 | -58 | -32 | 14 |
|  | Lateral occipital cortex | R | 19 | 545 | <.001 | 40 | -84 | -20 |
| 9 |  |  | 39 | 30 | <.001 | 54 | -64 | 20 |
|  | Occipital pole | L | 18 | 308 | <.001 | -28 | -88 | 4 |
|  | Lingual gyrus | L | 19 | 26 | <.001 | -20 | -60 | -4 |
|  | Temporal/occipital fusiform cortex | R | 19/37 | 59 | <.001 | 24 | -58 | -14 |
|  | Temporal fusiform cortex | L | 20 | 134 | <.001 | -34 | -6 | -50 |
|  | Middle temporal gyrus/angular gyrus | R | 21 | 134 | <.001 | 48 | -52 | 2 |
|  | Middle temporal gyrus | R | 21 | 27 | <.001 | 60 | -30 | -2 |
|  |  | L | 20 | 379 | <.001 | -50 | -14 | -38 |
|  |  |  | 37 | 37 | <.001 | 54 | -64 | -20 |
|  | Temporal pole | R | 38 | 71 | <.001 | 40 | 12 | -22 |
|  | Insula | L | 48 | 81 | <.001 | -44 | -10 | -4 |
|  |  |  | 20 | 27 | <.001 | -36 | -10 | -14 |
|  | Cerebellum crus I | R | - | 298 | <.001 | 50 | -58 | -30 |
|  |  | L | - | 42 | <.001 | -50 | -52 | -34 |
|  | crus II | L | - | 28 | <.001 | -44 | -68 | -54 |
|  |  | L | - | 25 | <.001 | -40 | -44 | -46 |
|  |  | R | - | 40 | <.001 | 20 | -90 | -38 |
|  | Lingual gyrus/Cerebellum crus II | L | - | 743 | <.001 | -22 | -90 | -40 |
|  | Cerebellum I-IV | R | - | 123 | <.001 | 14 | -44 | -34 |
|  | Cerebellum VI | L | - | 81 | <.001 | -26 | -72 | -22 |
|  | Cerebellum VIIIb/IX | R | - | 54 | <.001 | 10 | -62 | -60 |
|  | Cerebellum IX | L | - | 161 | <.001 | -4 | -46 | -54 |
|  | Thalamus | R | - | 53 | <.001 | 16 | -24 | 2 |
|  |  |  | - | 36 | <.001 | 16 | -14 | 14 |
|  | Fornix | L | - | 34 | <.001 | -4 | -12 | -8 |
|  |  | R | - | 41 | <.001 | 6 | -6 | -10 |
|  | Brain stem | R | - | 41 | <.001 | 8 | -24 | -12 |
|  |  | R | - | 28 | <.001 | 10 | -44 | -48 |
|  | Hippocampal complex | R | 47 | 378 | <.001 | 32 | -10 | -48 |
